# Nanocrystal-Loaded Micelles for the Enhanced In Vivo Circulation of Docetaxel

**DOI:** 10.3390/molecules26154481

**Published:** 2021-07-24

**Authors:** Meng Cheng, Qiaoming Liu, Tiantian Gan, Yuanying Fang, Pengfei Yue, Yongbing Sun, Yi Jin, Jianfang Feng, Liangxing Tu

**Affiliations:** 1 National Pharmaceutical Engineering Center for Solid Preparation in Chinese Herbal Medicine, Jiangxi University of Traditional Chinese Medicine, Nanchang 330006, China; cmjxzyy@126.com (M.C.); liuqm.to12@foxmail.com (Q.L.); gantt_1@126.com (T.G.); fangyuanying@163.com (Y.F.); ypfpharm@126.com (P.Y.); yongbing_sun@hotmail.com (Y.S.); jinyizju@hotmail.com (Y.J.); 2 School of Pharmacy, Guangxi University of Chinese Medicine, Nanning 530200, China

**Keywords:** nanocrystals, micelles, circulation, in vivo, docetaxel

## Abstract

Prolonging in vivo circulation has proved to be an efficient route for enhancing the therapeutic effect of rapidly metabolized drugs. In this study, we aimed to construct a nanocrystal-loaded micelles delivery system to enhance the blood circulation of docetaxel (DOC). We employed high-pressure homogenization to prepare docetaxel nanocrystals (DOC(Nc)), and then produced docetaxel nanocrystal-loaded micelles (DOC(Nc)@mPEG-PLA) by a thin-film hydration method. The particle sizes of optimized DOC(Nc), docetaxel micelles (DOC@mPEG-PLA), and DOC(Nc)@mPEG-PLA were 168.4, 36.3, and 72.5 nm, respectively. The crystallinity of docetaxel was decreased after transforming it into nanocrystals, and the crystalline state of docetaxel in micelles was amorphous. The constructed DOC(Nc)@mPEG-PLA showed good stability as its particle size showed no significant change in 7 days. Despite their rapid dissolution, docetaxel nanocrystals exhibited higher bioavailability. The micelles prolonged the retention time of docetaxel in the circulation system of rats, and DOC(Nc)@mPEG-PLA exhibited the highest retention time and bioavailability. These results reveal that constructing nanocrystal-loaded micelles may be a promising way to enhance the in vivo circulation and bioavailability of rapidly metabolized drugs such as docetaxel.

## 1. Introduction

Docetaxel (DOC), a typical taxane, has been approved as a first-line anti-tumor drug in clinical treatments [1,2]. Like other taxanes (paclitaxel, cabazitaxel, etc. [3]), docetaxel primarily operates its anti-tumor efficacy by disrupting the microtubular network, inducing a sustained block at the metaphase–anaphase boundary during cell division [4]. As a compound belonging to BCS IV, the only commercially available formulation containing docetaxel is intravenous injection (such as Taxotere^®^ and Docetaxel^®^), in which polysorbate 80 and dehydrated alcohol are used as cosolvents. Unfortunately, these cosolvents contribute to the majority of side reactions, including hypersensitivity, febrile neutropenia, fatigue, fluid retention, and peripheral neuropathy, that docetaxel injections produce, hence limiting its clinical application. Oral administration is the safest and most convenient administration route. Over the past decades, researchers have launched numerous strategies, such as nanoparticles [5,6], nano-emulsion [7], proniosomes [8], liposomes [9], and prodrugs [10], to enhance the oral bioavailability of docetaxel; however, low bioavailability still hinders the development of oral formulations for docetaxel. In this case, developing intravenous injections with higher safety and efficacy is an additional and promising strategy. Considering docetaxel is a rapidly metabolized drug, enhancing its in vivo circulation may benefit its therapeutic efficacy.

Micelles are self-assembled core-shell nanocarriers formed by surfactants or polymers. Considering their advantages, such as their ease of production, high drug loading (up to 30%), small particle sizes (below 200 nm), and ease of modification [11,12,13], micelles are a good choice for enhancing the safety and therapeutic efficacy of intravenous formulations [14]. Due to misunderstandings about the mechanism of micellar solubilization, micelles are sometimes regarded as the “solution” of the solutes, but this is not the case [15]. Hence, micelles can exhibit different pharmacokinetic performance to those of intravenous injection solutions. In general, due to their hydrophilic shell, micelles can signal the recognition of the reticuloendothelial system (RES), and thereby decrease the clearance ratio of the RES, resulting in a prolonged in vivo circulation [16,17]. To further enhance the in vivo circulation of micelles, researchers have employed various materials to modify the surface of micelles; one typical responsive is PEGylation [18,19]. With the aid of polyethylene glycol (PEG) and its derivatives, the hydrophilic profile of the micelle surface is improved [20], thus enhancing their blood circulation. However, in recent years, polymeric micelles have increasingly been formed by polymers synthesized from more than three different materials, with the increasing complexity of polymers raising concerns regarding the safety of materials and the commercial viability of these polymer micelles. These concerns have limited the application of the classical strategy (surface modification with materials) for enhancing in vivo circulation of micelles. Therefore, we hypothesized that combining micelles with other formulations that may improve blood circulation, may be a promising way to further enhance the in vivo circulation of micelles.

Nanocrystals, also called nanosuspension, is an additional technology for developing intravenous injection. Owing to its advantages such as high drug loading (up to 100%), ease of scale-up, and its organic, solvent-free preparation process (top-down method [21]), nanocrystals technology has exhibited great potential in numerous delivery systems. To date, there are over 10 products based on nanocrystals that have been approved by the FDA for oral administration (like Rapamune^®^, Emend^®^), and intramuscular injection (such as Invega Sustenna^®^ and Aristada^®^) [22,23,24,25]. Though some researchers have reported that some nanocrystals may not alter or improve the pharmacokinetic performance of drugs delivered via intravenous administration [26,27,28], a more common observation has been that nanocrystals may enhance the retention time and bioavailability of coarse drugs. As a foreign substance, nanocrystals are expected to be uptaken by the mononuclear phagocyte system (MPS) [29]; however, the internalization of nanocrystals in macrophages is limited (about 68% of ingested nanocrystals remained intact in macrophages 24 h post uptake [30,31]). The undegraded nanocrystals can be released intact with a similar size and shape to the original nanocrystals, hence achieving a prolonged in vivo circulation [32,33,34]. However, what should be borne in mind is that, as a pure drug system, few modifications can be made to nanocrystals, limiting the potential to further improve circulation time using functional materials. Embedding nanocrystals in another easily modified delivery system may improve their in vivo circulation.

**Figure 1:**
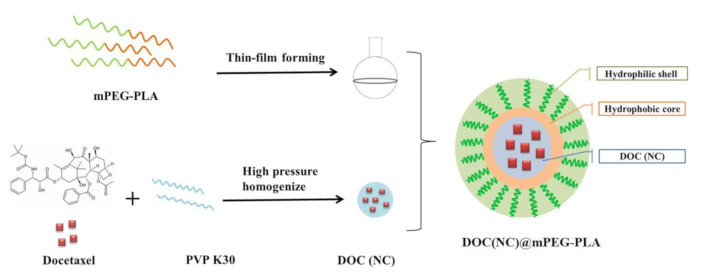
The preparation scheme of docetaxel nanocrystal-loaded micelles (DOC(Nc)@mPEG-PLA).

Considering the statements above, we aimed to construct a novel nanocrystal-loaded micelles delivery system to improve the in vivo circulation of docetaxel, docetaxel nanocrystals, and docetaxel micelles. Herein, we prepared docetaxel nanocrystals (DOC(Nc) by using high-pressure homogenization, and then embedded DOC(Nc) into the inner core of the micelles by using mPEG-PLA as their structural material. Figure 1 illustrates the preparation scheme of docetaxel nanocrystal-loaded micelles (DOC(Nc)@mPEG-PLA).

## 2. Results and Discussion

### 2.1. Preparation of DOC(Nc)@mPEG-PLA

**Figure 2:**
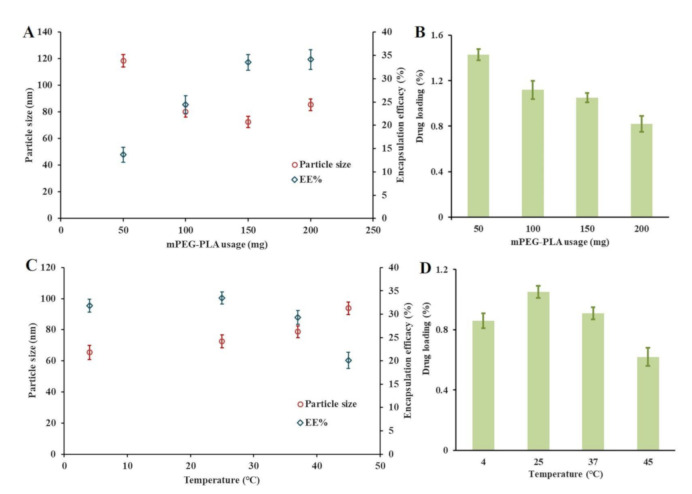
The effects of mPEG-PLA usage and hydration temperatures on the particle sizes, or encapsulation efficacy (**A**,**C**), and drug loading (**B**,**D**) of DOC(Nc)@mPEG-PLA. (*n* = 3).

To prepare fine docetaxel nanocrystal-loaded micelles (DOC(Nc)@mPEG-PLA) with small particle sizes, high encapsulation efficacy (EE), and drug loading (DL), we optimized the preparation parameters via mPEG-PLA usage and hydration temperatures, and the results are shown in Figure 2. Though drug loading was increased and mPEG-PLA usage was decreased (Figure 2B), the smallest particle size with high encapsulation efficacy was gained when the mPEG-PLA usage was 150 mg (Figure 2A); hence, we selected 150 mg as the usage of mPEG-PLA. Hydration temperature was another essential factor affecting the construction of micelles, and in this study, we set the hydration temperature at 4, 25, 37, and 45 °C. We observed that the particle sizes of DOC(Nc)@mPEG-PLA increased as hydration temperature increased, while the highest DL and EE were reached at 25 °C. Therefore, we selected 25 °C as the hydration temperature.

Combining the results shown above, we achieved the optimized preparation process of DOC(Nc)@mPEG-PLA, which can be described as follows: 150 mg mPEG-PLA is dispersed in 10 mL 95% ethanol, before a thin film is formed by removing the ethanol under 50 °C. Added to this film is 10 mL DOC(Nc) (containing 4 mg docetaxel), the film is then subject to sonication for 3 min, holding for 30 min at 25 °C.

### 2.2. Characterization of DOC(Nc)@mPEG-PLA

As shown in Table 1, The particle sizes of DOC(Nc) were 168.4 nm. After embedding docetaxel nanocrystals into the inner hydrophobic core of micelles, the particles sizes of docetaxel micelles (DOC@mPEG-PLA) were slightly increased from 36.3 nm to 72.5 nm. The DL and EE of DOC@mPEG-PLA were 4.89% and 76.75%, respectively, and those of DOC(Nc)@mPEG-PLA were 1.05% and 33.51%, respectively. The decreased DL and EE of nanocrystal-loaded micelles may be explained by the way nanocrystals may enhance the hydrophilicity of docetaxel, thus interrupting the entry process of nanocrystals into the inner hydrophobic core of micelles.

**Figure 3:**
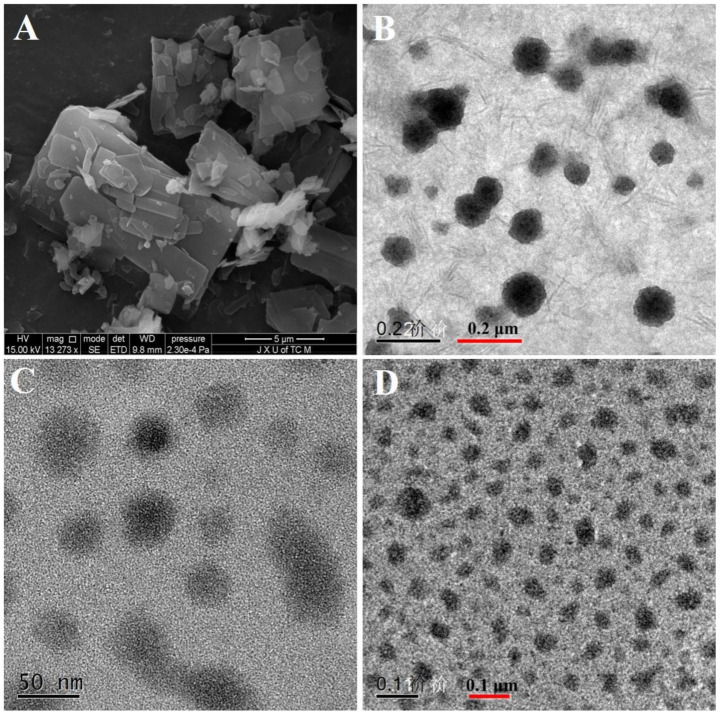
The SEM photo of DOC (**A**); the TEM photos of DOC(Nc) (**B**), DOC@mPEG-PLA (**C**), and DOC(Nc)@mPEG-PLA (**D**).

As shown in Figure 3A, the crude docetaxel had an irregular shape and its diameter was on the micron scale. After transferring the docetaxel into nanocrystals (DOC(Nc)) or micelles (DOC@mPEG-PLA), the morphology became more spherical (Figure 3B,C). Because of the embedding of nanocrystals, the diameter of micelles slightly increased, and the nanocrystal-loaded micelles (DOC(Nc)@mPEG-PLA) became more spherical (Figure 3D). The particle sizes of DOC(Nc), DOC(Nc)@mPEG-PLA, and DOC(Nc)@mPEG-PLA observed in TEM spectra were similar to that detected by Nano ZS 90 nanoparticle size and zeta potential meter (Malvern Instruments Co. Ltd., Malvern, UK).

**Figure 4:**
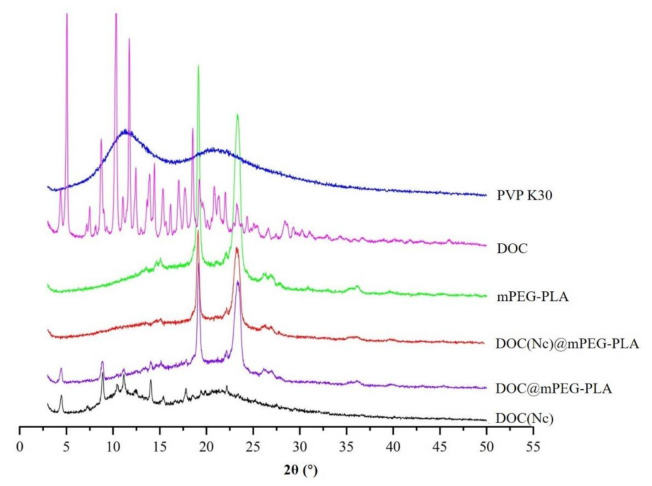
The XRD spectra of different materials.

In this study, we employed x-ray diffraction (XRD) (Ultima IV, Rigaku, Tokyo, Japan) and differential scanning calorimetry (DSC) (DSC Q2000, TA Instruments, New Castle, DE, America) to explore the crystal transition of docetaxel during the preparation processes of nanocrystals, micelles, and nanocrystal-loaded micelles. As shown in Figure 4, the coarse docetaxel is in a crystalline state, with its crystallinity drastically decreased in the production process of nanocrystals as there are only several weak diffraction peaks in the XRD spectra of DOC(Nc). Few diffraction peaks of docetaxel were left when transferring docetaxel to docetaxel micelles, revealing that DOC@mPEG-PLA was almost in an amorphous state. Excluding the influence of diffraction peaks of excipients (PVP K30 and mPEG-PLA), there are no obvious diffraction peaks of docetaxel in the XRD spectra of DOC(Nc)@mPEG-PLA, indicating the nanocrystal-loaded micelles were in amorphous state.

**Figure 5:**
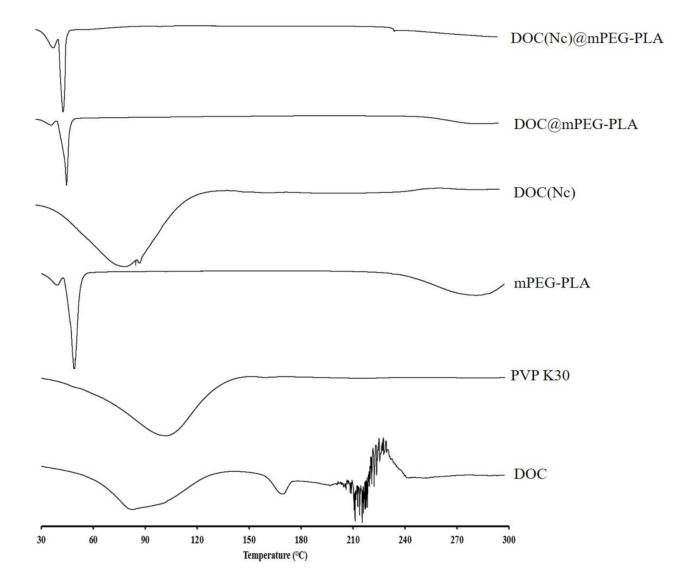
The DSC spectra of different materials.

The DSC spectra were drawn at scanning temperatures ranging from 30 to 300 °C with a scan rate of 10 °C/min. In the DSC spectra of docetaxel, endothermic peaks at about 220 °C can be observed; however, after treatments of preparation processes of nanocrystals, micelles, and nanocrystal-loaded micelles, these endothermic peaks are absent (Figure 5). Combining the results of the XRD and DSC spectra, it is certain that the docetaxel had undergone crystal transition in the preparation processes of the different formulations.

### 2.3. Release Profile of DOC(Nc)@mPEG-PLA

**Figure 6:**
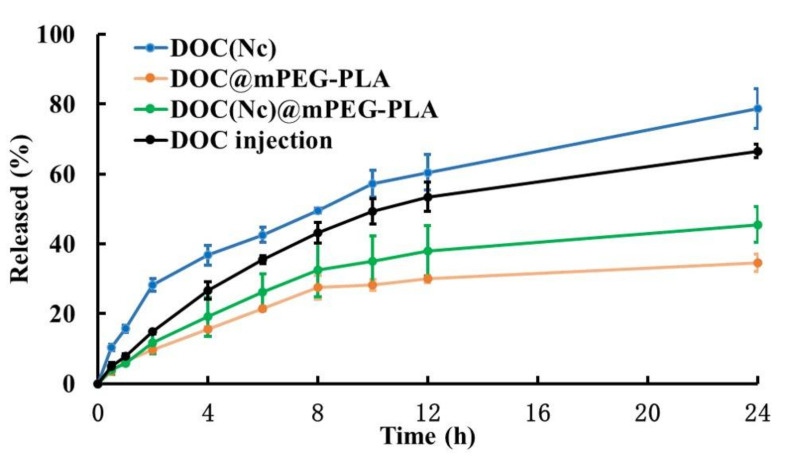
Release profiles of different formulations in a PBS buffer (pH 7.4) at 37 °C (*n* = 3).

As in vitro release profile typically has good correlation to in vivo performance, we operated a release study to better understand the in vivo pharmacokinetic behaviors of different formulations. The in vitro release behaviors of DOC, DOC(Nc), DOC@mPEG-PLA, and DOC(Nc)@mPEG-PLA were studied by dialysis performed in a PBS buffer (pH 7.4), which could be used to simulate the conditions in blood circulation. Owing to its decreased particle sizes and diffusion layer, DOC(Nc) released faster than DOC [35], and the release rate of DOC slowed down after transferring it into micelles, which may be attributed to factors such as hydrophobicity of the inner core of micelles and hydrogen bonding between molecules [36,37,38]. The release rate of DOC(Nc)@mPEG-PLA was faster than DOC@mPEG-PLA, and slower than DOC (Figure 6).

### 2.4. Stability of DOC(Nc)@mPEG-PLA

**Figure 7:**
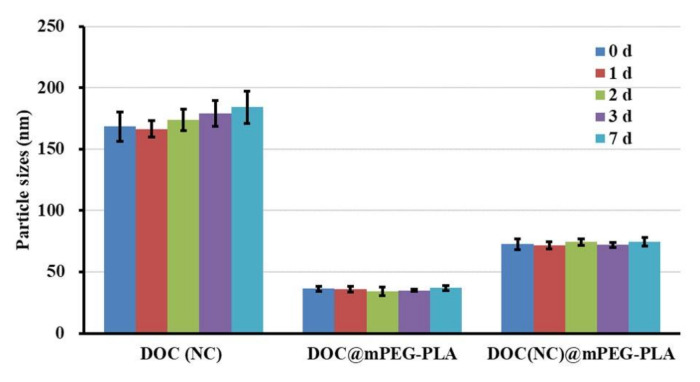
The particle sizes of DOC(Nc), DOC@mPEG-PLA, and DOC(Nc)@mPEG-PLA after holding at room temperature for 7 days. (*n* = 3).

After placing the DOC(Nc), DOC@mPEG-PLA, and DOC(Nc)@mPEG-PLA at room temperature for 7 days, the particle sizes of each sample at 0, 1, 2, 3, and 7 days were detected. Figure 7 illustrates that there was no significant change to particle sizes during the experimental period, revealing that these three formulations had good stability at room temperature without any dilution. In addition, after incubating with a PBS buffer or PBS buffer containing 10% FBS (10% FBS-PBS buffer) for 8 h, the particle sizes of DOC(Nc), DOC@mPEG-PLA, and DOC(Nc)@mPEG-PLA were changed from 173.5 ± 8.3, 33.8 ± 1.9, and 77.9 ± 3.3 to 176.4 ± 5.3, 30.7 ± 2.8, and 75.2 ± 4.9, respectively, in PBS buffer, while in 10% (*v/v*) FBS-PBS buffer, the particle sizes were changed from 191.4 ± 15.3, 42.8 ± 1.7, and 83.5 ± 5.0 to 382.5 ± 21.4, 57.2 ± 3.8, and 101.3 ± 6.7 for DOC(Nc), DOC@mPEG-PLA, and DOC(Nc)@mPEG-PLA, respectively. These results show that these nano formulations were stable in a PBS buffer; however, after incubating with serum, the particle sizes of nanocrystals largely increased, which may be due to the drug-protein interaction [39], and, due to the excellent hydrophilic property of mPEG, the micelles and nanocrystal-loaded micelles exhibited good stability [40].

### 2.5. Pharmacokinetic Behavior of DOC(Nc)@mPEG-PLA

**Figure 8:**
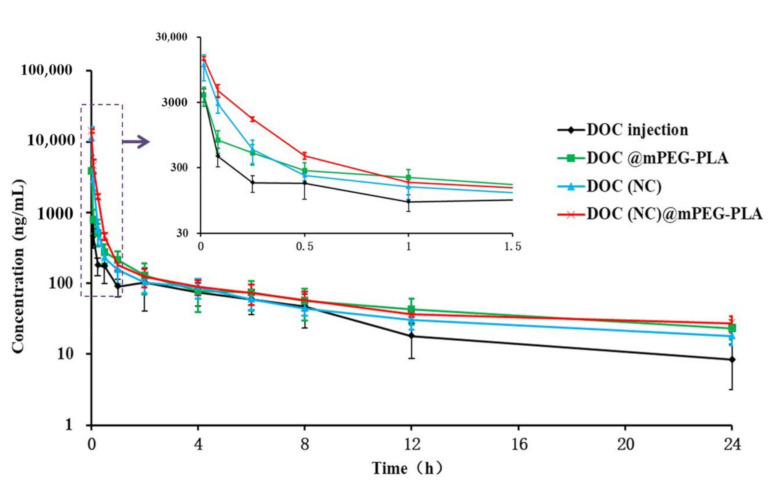
The concentration–time curves of different formulations on rats via intravenous route at 10 mg/kg (*n* = 5).

It is certain that the pharmacokinetic behavior of DOC changed, as shown in Table 2 and Figure 8. The C_1min_ of DOC injection (Taxotere^®^) was 3947 ng/mL, while that for DOC (NC) was 11,181 ng/mL. In addition, the bioavailability of DOC (NC) was higher than that of DOC injection. The enhanced C_1min_ and bioavailability of DOC (NC) can be explained by nanocrystals being uptaken by the reticuloendothelial system (RES) [41], preventing the rapid metabolization of DOC by the liver and allowing some nanocrystals to escape from the RES without being internalized [30]. The C_1min_ of DOC injection and DOC@mPEG-PLA were similar; however the bioavailability of DOC was enhanced by micelles, which may be caused by their sustained release and their capacity to escape the RES [42,43,44]. DOC(NC)@mPEG-PLA exhibited the highest C_1min_ and bioavailability, which were 3.58- and 2.69-fold, respectively, to DOC injection. In addition, compared to DOC injection, DOC (NC), and DOC@mPEG-PLA, DOC(NC)@mPEG-PLA showed decreased CL and enhanced MRT and t_1/2_. These results reveal the in vivo performance of DOC(NC)@mPEG-PLA: firstly, the docetaxel nanocrystals underwent sustained release from DOC(NC)@mPEG-PLA; subsequently, DOC(NC) was uptaken by the RES; before finally, the DOC(NC) excited the RES by diffusing down the drug concentration gradient. With the enhanced blood circulation of nanocrystals and the sustained-release behavior of micelles, the nanocrystal–micelles system showed the largest potential for enhancing in vivo circulation and bioavailability of rapidly metabolized drugs such as docetaxel.

Owing to the ease of modifying its surface behavior, micelles, modified with multiple functional agents, may enhance the in vivo performance of docetaxel, such as when it is modified by monomethoxy poly(ethylene glycol)-poly(epsilon-caprolactone) (mPEG-PCL) [45] and stearic acid (SA)-modified Bletilla striata polysaccharides (BSPs) copolymers [46]. However, studies have primarily focused on how to enhance targeting efficacy and cell/tissue penetration ability, while attempts to fabricate multi-nanoparticle embedded complexes to enhance the in vivo circulation have been crude. As for nanocrystals, several researchers have attempted to enhance the in vivo performance of docetaxel nanocrystals, for example, after modification with apo-Transferrin human (Tf) [47] or trans-activator of transcription (TAT) peptide [48], the anti-tumor efficacy of unmodified docetaxel micelles was enhanced. However, what needs to be emphasized is that, as a pure drug system, modification of nanocrystals is limited. Hence, with the construction of nanocrystal micelles, we can use the enhanced in vivo circulation ability of nanocrystals to enhance the circulation time of micelles, and can similarly take the advantages of micelles (such as enhanced circulation time and ease of modification) to improve the in vivo performance of nanocrystals.

## 3. Materials and Methods

### 3.1. Materials

Docetaxel (purity > 99%) and paclitaxel (purity > 99%) were purchased from Wuhan Zeshancheng Biotechnology Co., Ltd., Wuhan, China. Povidone K30 (PVP K30) was purchased from Boai NKY Pharmaceuticals Ltd., Jiaozuo, China. Methoxy polyethylene glycol-b-poly(L-lactide) (mPEG-PLA) was supplied by Sigma-Aldrich, City of Saint Loui, USA. Phosphate-buffered saline (PBS) was gained from Aladdin, Shanghai, China. Fetal bovine serum (FBS) was gained from Gibco, New York, USA. All other materials and reagents were of analytical grade and purified water was used throughout this study.

Male Sprague-Dawley (SD) rats, weighing 250 ± 20 g, were supplied by the Hunan STJ Laboratory Animal Co., Ltd. (Changsha, China).

### 3.2. Preparation of Docetaxel Nanocrystals (DOC(Nc))

The docetaxel nanocrystals were prepared via high-pressure homogenization technology. We dispersed 20 mg docetaxel in 50 mL purified water (containing 100 mg PVP K30), and then pre-treated by a high shear homogenizer (Fluko^®^ FA25, FLUKO, Shanghai, China) at 13,000 rpm for 10 min. The crude suspension was treated in a high-pressure homogenizer (AH NANO, ATS, Shanghai, China) thereafter. The docetaxel nanocrystals were harvested after homogenizing at 1000 bar for 20 cycles.

### 3.3. Preparation of Docetaxel Micelles (DOC@mPEG-PLA)

The docetaxel micelles were prepared by using a thin-film hydration method. Briefly, 10 mg docetaxel and 150 mg mPEG-PLA were dispersed in 10 mL 95% ethanol, and the ethanol was removed by evaporation under 50 °C to form a thin film, which was hydrated by 10 mL purified water under sonication for 3 min and holding for 30 min at 25 °C. The docetaxel micelles were gained after filtering the unencapsulated DOC by using 0.22 µm microporous membrane.

### 3.4. Preparation of Docetaxel Nanocrystals Loaded Micelles (DOC(Nc)@mPEG-PLA)

Several mPEG-PLA were dispersed in 10 mL 95% ethanol, and then the thin film was formed by removing the ethanol under 50 °C. 10 mL DOC(Nc), prepared as Section 3.2., was added to the thin film, and underwent sonication for 3 min, holding for 30 min at pre-set temperatures. The unencapsulated DOC(Nc) was removed by centrifugation at 12,000 rpm for 10 min, followed by filtration through 0.22 µm microporous membrane. The effects of mPEG-PLA usage and hydration temperatures on the particle sizes, DL, and EE of DOC(Nc)@mPEG-PLA were studied.

### 3.5. Characterization of DOC(Nc)@mPEG-PLA

#### 3.5.1. Particle Sizes

The particle sizes of DOC(Nc), DOC@mPEG-PLA, and DOC(Nc)@mPEG-PLA were characterized by Nano ZS 90 nanoparticle size and zeta potential meter (Malvern Instruments Co., Ltd., Malvern, UK), and all samples were analyzed in triplicate.

#### 3.5.2. Drug Loading and Encapsulation Efficacy

The drug loading (DL) and encapsulation efficiency (EE) of DOC@mPEG-PLA and DOC(Nc)@mPEG-PLA were measured as reported prior [49,50]. In brief, 2 mL methanol was added to 100 µL DOC@mPEG-PLA or DOC(Nc)@mPEG-PLA by vortexing for 1 min to destroy the structure of the micelles, and then the samples were centrifugated at 12,000 rpm for 10 min. The drug concentration of the supernatant was detected by HPLC which was performed as follows: employing Ultimate XB-C18 column (4.6 × 250 mm, 5 µm) as the detection column, selecting acetonitrile-water solution (52:48) as mobile phase, and operating the detection at 30 °C with flow rate and detection wavelength at 1 mL/min and 230 nm, respectively. The HPLC analysis was validated and met the methodological requirements.

The drug loading (*DL*) and encapsulation efficiency (*EE*) of DOC@mPEG-PLA and DOC(Nc)@mPEG-PLA were calculated as below:

$$DL=\frac{Amount\;of\;DOC\;loaded}{Total\;amout\;of\;DOC\;and\;excipients\;loaded\;}\times 100\%\;EE=\frac{Amount\;of\;DOC\;loaded}{Amout\;of\;DOC\;used\;}\times 100\%$$

#### 3.5.3. Particle Morphology

The morphology of docetaxel was performed on a scanning electron microscope (SEM) (Quanta 250, FEI, Hillsboro, OR, USA), after coating the surface of the docetaxel particles with gold. After staining, the samples were fixed on a copper mesh (Ted Pella, Redding, CA, USA) by phosphotungstic acid, the morphology of DOC(Nc), DOC@mPEG-PLA, and DOC(Nc)@mPEG-PLA was observed by using a transmission electron microscope (TEM) (Tecnai Spirit, FEI, Hillsboro, OR, USA).

#### 3.5.4. Crystalline Study

The crystalline states of DOC, DOC(Nc), DOC@mPEG-PLA, and DOC(Nc)@mPEG-PLA were studied by employing x-ray diffraction (XRD) (Ultima IV, Rigaku, Tokyo, Japan) and differential scanning calorimetry (DSC) (DSC Q2000, TA Instruments, New Castle, DE, America).

### 3.6. Release Behaviors In Vitro

The in vitro release behaviors of DOC, DOC(Nc), DOC@mPEG-PLA, and DOC(Nc)@mPEG-PLA were studied by dialysis [35,51]. Firstly, the DOC, DOC(Nc), DOC@mPEG-PLA, and DOC(Nc)@mPEG-PLA were diluted to 100 μg/mL (calculated as docetaxel) with PBS buffer (pH 7.4), and then 2 mL diluted samples was added into dialysis bags (3000 Da molecular weight cutoff). After immersing these analysis bags into a 30 mL PBS buffer with stirring speed of 100 rpm at 37 °C, 0.5 mL samples were harvested at 0.5, 1, 2, 4, 6, 8, and 12 h. Considering the solubility of docetaxel in water is about 3.9–6.0 μg/mL [52,53], the release medium was replaced by fresh PBS buffer at each pre-determined sampling time point to maintain sink conditions [54]. The DOC concentration in the release buffer was detected via HPLC as exhibited in Section 3.5.2.

### 3.7. Stability Evaluation

The stability of different formulations was studied in this study. Briefly, freshly prepared DOC(Nc), DOC@mPEG-PLA, and DOC(Nc)@mPEG-PLA (without dilution) were performed under room temperature for 0, 1, 2, 3, and 7 days, and the particle sizes of all samples collected at pre-determined times were analyzed in triplicate. In addition, we evaluated the stability of nanoparticles in a PBS buffer and 10% (*v*/*v*) FBS-PBS buffer after diluting fresh prepared nanoparticles to 100 μg/mL (calculated as docetaxel) and holding at 37 °C for 8 h [55].

### 3.8. In Vivo Pharmacokinetic Studies

We employed male Sprague-Dawley (SD) rats to explore the in vivo pharmacokinetic performance of different formulations. Briefly, twenty SD rats, weighing 250 ± 20 g, were randomly divided into four groups, and intravenously injected with docetaxel injection (prepared similarly to Taxotere^®^; in brief, 80 mg docetaxel was dissolved in 2 mL polysorbate 80 80, and then dispersed in 6 mL 13%(*w*/*w*) dehydrated alcohol), DOC(Nc), DOC@mPEG-PLA, and DOC(Nc)@mPEG-PLA at 10 mg/kg. After administration for 1, 5, 15, and 30 min; and 1, 2, 4, 6, 8, 12, and 24 h, a blood sample of about 0.3 mL was collected by retro-orbital puncture. The plasma was centrifuged at 4000 rpm for 10 min at 4 °C and stored at −20 °C until analysis thereafter.

Before pumping the samples into an HPLC-MS system for concentration detection, the plasma samples were treated as follows: 50 μL plasma samples, 10 μL paclitaxel solution (internal standard, 20 μg/mL), and 60 μL 0.3% formic acid-acetonitrile solution were mixed by vortexing for 3 min, and then centrifugated at 12,000 rpm for 10 min. The concentration of the supernatant was detected by HPLC-MS/MS performed as below:

Column: Ultimate AQ-C18 column (2.1 × 100 mm, 1.8 µm)

Mobile phase: 0.1% formic acid-acetonitrile solution (A) and 0.1% formic acid-water solution (B)

Gradient sequence: 0.1~3.0 min, 55~95% B; 3.0~3.1min, 95~55% B; 3.1~6.0 min, 55%B; at flow rate of 0.3 mL/min.

MS condition: Turbo ion spray interface voltage, −4.5 kV; turbo heater temperature, 500 °C; atomizing gas pressure, 340.5 kPa; auxiliary gas pressure, 345.5 kPa; curtain gas pressure, 205.0 kPa; decluster voltage, 100 V; collision voltage of docetaxel and paclitaxel, 37 eV and 20 eV; m/z, 830.37→549.24 for docetaxel and 876.36→286.3 for paclitaxel.

### 3.9. Statistical Analysis

Pharmacokinetic parameters were calculated using the DAS 3.2.8 pharmacokinetics program (developed by the Clinical Trial Center of Shanghai University of Traditional Chinese Medicine, Shanghai, China). All values are expressed as the means ± SD. The statistical analysis was performed using one-way ANOVA with SPSS Statistics 22.0 (SPSS Inc., Chicago, IL, USA). The differences were considered significant at *p* < 0.05.

## 4. Conclusions

In this study, we constructed docetaxel nanocrystals (DOC(Nc) with a diameter of 168.4 nm by HPH. We also prepared docetaxel micelles (DOC@mPEG-PLA) and docetaxel nanocrystal-loaded micelles (DOC(Nc)@mPEG-PLA), with particle sizes of 36.3 nm and 72.5 nm, respectively, by a thin-film hydration method. The results of XRD and DSC showed that the crystallinity of docetaxel decreased after transforming it into nanocrystals, and the docetaxel in DOC@mPEG-PLA and DOC(Nc)@mPEG-PLA were amorphous. The formulations constructed in this paper exhibited good stability in the 7 day stability study period. The bioavailability of docetaxel was enhanced by DOC(Nc) and DOC@mPEG-PLA, while DOC(Nc)@mPEG-PLA had the highest bioavailability, which was 2.69-, 1.39-, and 1.69-fold to DOC injection, DOC(Nc), and DOC@mPEG-PLA, respectively. In addition, compared to DOC injection, DOC (NC), and DOC@mPEG-PLA, DOC(NC)@mPEG-PLA showed decreased CL and enhanced MRT and t_1/2_. These results showed that DOC(NC)@mPEG-PLA exhibited the optimum pharmacokinetic behavior, and revealed that constructing nanocrystal-loaded micelles is a promising way to enhance the in vivo circulation and bioavailability of rapidly-metabolized drugs like docetaxel.

## Figures and Tables

**Table 1:** The particle sizes, drug loading (DL), and encapsulation efficiency (EE) of different formulations. (mean ± SD, *n* = 3).

	Sizes (nm)	DL (%)	EE (%)
DOC(Nc)	168.4 ± 11.9	N/A	N/A
DOC@mPEG-PLA	36.3 ± 2.0	4.89 ± 0.07	76.75 ± 0.62
DOC(Nc)@mPEG-PLA	72.5 ± 4.2	1.05 ± 0.04	33.51 ± 1.70

**Table 2:** Pharmacokinetics parameters of different formulations on rats via intravenous route at 10 mg/kg (mean ± SD, *n* = 5).

Parameters	Units	DOC Injection	DOC (NC)	DOC@mPEG-PLA	DOC(NC)@mPEG-PLA
AUC(0→∞)	ng/L h	1313 ± 342	2542 ± 405 *	2084 ± 517 *	3532 ± 157 *^,#,†^
MRT(0→∞)	h	7.197 ± 3.436	5.545 ± 2.185	10.387 ± 2.142 *	15.668 ± 2.520 *^,#,†^
t_1/2_	h	7.334 ± 4.212	7.886 ± 3.831	9.945 ± 2.627	17.504 ± 3.362 *^,#,†^
CL	L/h/Kg	0.008 ± 0.003	0.004 ± 0.001 *	0.005 ± 0.002	0.003 ± 0.001 *^,†^
C_1min_	ng/L	3947 ± 914	11,181 ± 4741 *	3892 ± 1274	14,120 ± 845 *^,†^

* *p* < 0.05 vs. DOC injection, ^#^ *p* < 0.05 vs. DOC (NC), ^†^ *p* < 0.05 vs. DOC@mPEG-PLA.

## Data Availability

The data used and/or analyzed during this study are available from the corresponding author upon reasonable request.

## Sample Availability

Samples of the formulations are available from the corresponding authors.
